# Maternal alcohol use during pregnancy in a general national population in South Africa

**DOI:** 10.4102/sajpsychiatry.v25i0.1236

**Published:** 2019-01-17

**Authors:** Karl Peltzer, Supa Pengpid

**Affiliations:** 1 HIV/AIDS/STIs and TB (HAST), Human Sciences Research Council, South Africa; 2 Department of Research Innovation and Development, University of Limpopo, South Africa; 3 ASEAN Institute for Health Development, Mahidol University, Thailand

## Abstract

**Objective:**

Alcohol use in pregnancy is linked with various negative health effects on the infant. The aim of this study was to examine the prevalence of maternal alcohol use during pregnancy and socio-demographic and health correlates.

**Methods:**

Data of ever-pregnant women from the cross-sectional ‘South African National Health and Nutrition Examination Survey (SANHANES-1) 2011–2012’ were analysed. The sample included 5089 adolescents and adult women aged 15–55 years. They responded to questions on alcohol use, socio-demographic and health indicators.

**Results:**

The results indicated that 3.7% (95.0% confidence interval [CI] = 3.1, 4.5) of South African women had engaged in alcohol use during their pregnancy. In adjusted analysis, being mixed race, not employed, poor self-rated health status, ever been diagnosed with tuberculosis and having partial post-traumatic stress disorder were found to be associated with alcohol use during pregnancy.

**Conclusions:**

The study findings suggest links between socio-demographic and health variables and prenatal alcohol use, which may have public health policy implications.

## Introduction

Alcohol use in pregnancy is associated with negative health effects on the infant, such as lifelong disabilities, known as foetal alcohol spectrum disorders.^1,2^ Globally, in the general population, the estimated prevalence of alcohol use during pregnancy was 9.8%,^1^ and in Southern Africa this figure was 6.6%, including South Africa (13.2%).^3^ These estimates are largely based on local surveys, which ranged (e.g. for South Africa) from 3.2%^4^ and 6.5%^5^ to 20.4%^6^ and 42.8%.^7^ There is a lack of national data on alcohol use during pregnancy in South Africa.

Correlates of alcohol use during pregnancy include socio-demographic factors such as women of older age, having a higher income, being employed or unemployed, and ethnicity or population group (mixed race women).^8,9,10^ Moreover, smoking,^11,12^ traumatic experiences,^13^ exposure to violence^9^ and poor physical and mental health^9,14^ have been identified as risk factors for maternal alcohol use during pregnancy. There is a lack of studies investigating physical and mental correlates of maternal alcohol use during pregnancy. Understanding these correlates could lead to the development of more effective prevention strategies for maternal alcohol use.^9^

The aim of this study was to examine the prevalence of maternal alcohol use during pregnancy and socio-demographic and health correlates.

## Methods

### Sample and procedure

The ‘South African National Health and Nutrition Examination Survey (SANHANES-1)’ is a cross-sectional and multi-stage population-based household survey conducted in 2011–2012; in more detail it is discussed elsewhere.^15^ A total of 25 532 participants (92.6%) completed the interview.^15^

### Measures

Socio-demographic data included age, sex, employment status, population group, province and residential status.

Maternal alcohol use during pregnancy was measured through the following questions: (1) ‘During pregnancy, did you ever have a drink containing alcohol?’ (yes, no) and (2) ‘during pregnancy, how many drinks containing alcohol did you have per day?’ (Responses ranged from 1 = 1 or 2 per week to 8 = 10 or more per day.)^15^ In addition, *risky or* hazardous drinking was assessed with the three-item ‘Alcohol Use Disorders Identification Test-Consumption (AUDIT-C)’. Total scores range from 0 to 12, with a score of 3 or more indicating risky or hazardous drinking or active alcohol use disorders^16^ (Cronbach’s alpha 0.89).

*Maternal tobacco use during pregnancy* was assessed using the following question: ‘during pregnancy, did you ever smoke tobacco or use any tobacco products?’ (yes, no). In addition, current daily tobacco use was also assessed.^16^

Self-rated health was assessed using the following question: ‘in general, how would you rate your health today?’^15^ Responses were dichotomised into as having ‘good health’ (1: very good, or 2: good) and ‘poor health’ ( 3: moderate, 4: bad or 5: very bad).

Chronic conditions were measured with the question:

has a doctor, nurse or health worker at a clinic or hospital told you that you have had any of the following conditions: high blood pressure, stroke, heart disease, heart attack or angina (chest pain), high blood cholesterol, high blood sugar or sugar diabetes?^15^

In addition, the participants were asked if they ever had been diagnosed with tuberculosis (yes or no).^15^

To assess the *experience of trauma events,* the participants were asked the following question:

Have you ever experienced any of the following events (14 events, e.g., ‘severe automobile accidents’ and ‘learned about the sudden, unexpected death of a family member or a close friend’)? (yes or no)^15^

Post-traumatic stress disorder (PTSD) was measured with the ‘Davidson Trauma Scale (DTS)’.^17^ Partial PTSD was defined as having at least one PTSD symptom from each of the three PTSD symptom clusters^18^ (Cronbach’s alpha 0.94).

Insomnia was measured with two items:

1. on the severity of nocturnal sleep problems: overall in the last 30 days, how much of a problem did you have with sleeping, such as falling asleep, waking up frequently during the night, or waking up too early in the morning?
2. and the severity of difficulty with daytime functioning overall in the last 30 days, how much of a problem did you have due to not feeling rested and refreshed during the day (e.g. feeling tired or not having energy)?

Response options ranged from 0 = none to 4 = extreme/cannot do^19^ (Cronbach’s alpha 0.82). Insomnia symptoms were classified as having total scores of ≥ 4–8.

Psychological distress was assessed with the 10-item Kessler questionnaire,^20^ which has been validated in South Africa^21^ (response options: 1 = never to 5 = all the time). Total scores of 30 or more indicate severe psychological distress^20^ (Cronbach’s alpha 0.93).

### Data analysis

Data were analysed using STATA software version 13.0 (Stata Corporation, College Station, TX, USA). Pearson’s chi-square statistics were used to test the differences in proportions. Multivariable logistic regression was used to compute the odds ratios (with 95.0% confidence interval [CI]) to determine the associations between socio-demographic and health characteristics and maternal alcohol use during pregnancy. Independent variables found significant with the outcome (alcohol use during pregnancy) in bivariate analysis were subsequently included in the multivariable regression model. Current alcohol and tobacco use and tobacco use during pregnancy were excluded because of collinearity. No further collinearity was identified. All models were adjusted for the multi-stage sampling design.

## Ethical consideration

Participants provided information on socio-demographic and health variables in face-to-face interviews after their informed consent was obtained. The current study sample is restricted to women who responded that they had ever been pregnant and were 15–55 years old (*N* = 5089). The study was approved by the research ethics committee (REC) of the Human Sciences Research Council (REC 6/16/11/11).

## Results

### Sample characteristics

The total sample included 5089 women who had been pregnant and were 15 to 55 years old, with a median age of 35.0 years (interquartile range [IQR] = 15) from South Africa. The majority of participants (80.0%) belonged to the black African population group, 39.6% were employed and 63.7% were residing in urban areas. About one in five of the participants (23.3%) rated their health as poor, 25.2% had one or more chronic conditions, 7.0% had ever been diagnosed with tuberculosis, 20.2% had experienced one or more traumatic events and 4.4% had a partial PTSD. In total, 7.4% of participants reported insomnia symptoms, 2.3% reported severe psychological distress, 4.8% told they had been using tobacco during their pregnancy, 9.1% told they were currently using tobacco daily and 11.9% were reported to be hazardous or harmful alcohol users.

Overall, 3.7% had been using alcohol when they were pregnant: 8.4% among the mixed race population group, 7.3% in the Free State, 7.2% in the Northern Cape and 6.1% in the Western Cape province. In bivariate analysis, it was found that the prevalence of alcohol use during pregnancy was higher among unemployed than among employed participants, in participants with poorer self-rated health status, having ever been diagnosed with tuberculosis, having traumatic stress, partial PTSD and psychological distress. About one in four (26.1%) of prenatal alcohol users had also been using tobacco during pregnancy, and 13.5% of prenatal alcohol users were current daily tobacco users and 19.7% were hazardous or harmful alcohol users (see Table 1).

**TABLE 1:** Sample characteristics and weighted prevalence of alcohol use during pregnancy.

Variable	Sample		Alcohol use during pregnancy				Chi-square
	*N*	%	%	95%	CI		*p*
**Socio-demographic**						
**Age (years)**						
All	5089	-	3.7	3.1	4.5		0.113
15–24	862	15.0	5.2	3.6	7.5		
25–34	1451	32.9	3.2	2.3	4.5		
35–55	2776	52.2	3.6	2.8	4.6		
**Population group**						
Black African	3457	80.0	3.2	2.5	4.1		0.003
White people	198	8.1	2.2	0.7	6.2		
Mixed race	1036	9.8	8.4	6.2	11.4		
Indian or Asian	343	2.1	4.7	1.2	16.6		
**Province**						
Western Cape	753	11.7	6.1	4.1	8.9		< 0.001
Eastern Cape	524	11.3	3.2	1.9	5.6		
Northern Cape	332	2.4	7.2	4.3	11.7		
Free State	305	5.4	7.3	4.6	11.2		
KwaZulu-Natal	802	18.5	3.3	2.0	5.5		
North West	619	6.7	3.2	1.8	5.8		
Gauteng	890	25.4	3.3	2.0	5.6		
Mpumalanga	473	8.0	2.0	0.9	4.2		
Limpopo	400	10.4	2.2	1.0	4.8		
**Employment status**						
Unemployed	3132	60.4	4.6	3.7	5.8		0.005
Employed	1860	39.6	2.2	1.5	3.1		
**Residence**						
Rural	1656	36.3	2.9	2.0	4.0		0.170
Urban	3132	63.7	4.2	3.3	5.3		
**Health variables**						
**Self-rated health status**						
Very good, good	3856	76.7	3.0	2.4	3.9		< 0.001
Moderate, bad, very bad	1173	23.3	5.8	4.3	7.8		
**Chronic conditions**						
None	3515	74.8	3.3	2.6	4.2		0.223
One or more	1300	25.2	5.0	3.7	6.6		
**Ever diagnosed with TB**						
No	4636	93.0	3.4	2.8	4.3		< 0.001
Yes	343	7.0	7.0	4.5	10.7		
**Traumatic stress**						
None	4021	79.8	3.2	2.5	4.0		0.009
One or more	899	20.2	6.0	4.2	8.4		
**PTSD**						
None	4824	95.6	3.5	2.8	4.2		< 0.001
Partial	197	4.4	9.6	6.0	15.1		
**Insomnia**						
0–3	4647	92.6	3.5	2.8	4.2		0.128
4–8	359	7.4	6.2	3.4	11.0		
**Psychological distress**						
< 30	4808	97.7	3.5	2.9	4.3		0.006
30 or more	127	2.3	11.5	6.2	20.4		
**Tobacco use during pregnancy**						
No	4638	95.2	2.7	2.1	3.4		< 0.001
Yes	419	4.8	26.1	20.5	32.6		
**Current tobacco use**						
None < daily	4331	90.9	2.8	2.2	3.6		< 0.001
Daily	660	9.1	13.5	10.3	17.4		
**Current alcohol use**						
Not hazardous or harmful	4328	88.1	1.5	1.1	2.0		< 0.001
Hazardous or harmful	679	11.9	19.7	15.8	24.3		

CI, confidence interval; PTSD, post-traumatic stress disorder; TB, tuberculosis.

Among women reporting alcohol use during pregnancy, 59.1% reported consuming alcohol once or twice per week, 23.7% reported three or four times per week, 7.8% reported five or six times per week, 5.4% stated once or twice per day and 4.1% stated three times or more per day.

### Associations with maternal alcohol use during pregnancy

In adjusted analysis, being mixed race, unemployed, poor self-rated health status, having ever been diagnosed with TB and having partial PTSD were found to be associated with alcohol use during pregnancy (see Table 2).

**TABLE 2:** Multivariable logistic regression on alcohol use during pregnancy.

Variable	AOR (95% CI)	*p*
**Socio-demographic**		
**Population group**		
Black African	1 (Reference)	
White people	1.64 (0.71, 3.78)	0.246
Mixed race	2.91 (1.94, 4.36)	<0.001
Indian or Asian	1.21 (0.34, 4.31)	0.763
**Employment status**		
Employed	1 (Reference)	
Not formally employed	1.78 (1.15, 2.74)	0.009
**Health variables**		
**Self-rated health status**		
Very good, good	1 (Reference)	
Moderate, bad, very bad	1.61 (1.09, 2.39)	0.017
**Ever diagnosed with TB**		
No	1 (Reference)	
Yes	1.94 (1.20, 3.14)	0.007
**Traumatic stress**		
None	1 (Reference)	
One or more	1.32 (0.83, 2.09)	0.245
**PTSD**		
None	1 (Reference)	
Partial	2.02 (1.12, 3.66)	0.020
**Psychological distress**		
< 30	1 (Reference)	
30 or more	1.64 (0.77, 3.47)	0.198

AOR, adjusted odds ratio; CI, confidence interval; PTSD, post-traumatic stress disorder; TB, tuberculosis.

## Discussion

To our knowledge, this is the first population-based national study assessing the prevalence of maternal alcohol use during pregnancy in South Africa. The study found a prevalence of 3.7% of maternal alcohol use during pregnancy in South Africa, which is lower than previous estimates based on local surveys in South Africa (13.2%), Southern Africa (6.6%) and globally (9.8%).^1,3^

Compared to this national survey in South Africa, the previously reported higher rate may be attributed to local surveys targeting higher risk groups such as the mixed race population groups. In addition, for this study the reference period for recalling alcohol use during pregnancy was the participants’ whole reproductive period, while the other studies recalled alcohol use only during their current pregnancy. A shorter recall reference period may produce a higher prevalence of alcohol use. Furthermore, the study found that the proportion of pregnant women who engaged in binge drinking (three or more drinks per day) during pregnancy out of all pregnant women who consumed any amount of alcohol was 4.3%, which seems similar to previous estimates for South Africa (< 5%) and lower than in 65 of 162 countries (> 25.0%).^22^ Binge drinking during pregnancy is a direct cause of the foetal alcohol syndrome (FAS) and therefore is of particular concern.^22^

In agreement with previous studies,^8,9,10,11,12,14^ this study found that demographic characteristics (e.g. being from the mixed race group and unemployed), tobacco use, poor self-rated health status, having had tuberculosis (TB) and partial PTSD increased the risk of maternal alcohol use during pregnancy. These risk groups should be specifically targeted for preconception alcohol prevention intervention.^22^ Furthermore, among the nine provinces in South Africa, the study found a particularly high prevalence of maternal alcohol use during pregnancy (6.1% – 7.3%) in three provinces (Free State, Northern Cape and Western Cape). Most of these three provinces have the highest proportion of mixed race population groups in South Africa, emphasising the need to target the mixed race female population with preconception alcohol prevention intervention.

The co-occurrence between tobacco and alcohol use is well established.^9^ The fact that both substances (alcohol and tobacco) can have negative effects on the foetus; therefore, it is of great importance to consider both in the assessment and intervention strategies.^9^ The association found between poor mental health (partial PTSD, and psychological distress in bivariate analysis) and prenatal alcohol use may be explained by the comorbidity with common mental disorders such as anxiety and depression,^9^ whereby self-medication with alcohol use may be one of the mechanisms.^9^ The association between TB and alcohol use in general has been established, with the latter being a risk factor for the former.^23^ As this study could not determine the direction of the relationships because of its cross-sectional nature, longitudinal studies are needed to clarify this.

## Study limitations

The study variables of maternal alcohol use during pregnancy were assessed retrospectively over possibly long periods, which may have introduced a recall bias. The self-report of prenatal alcohol use is probably a large underestimate.^24^ Furthermore, the cross-sectional nature of the study limits our ability to establish causality.

## Conclusion

This study found a prevalence rate of 3.7% of maternal alcohol use during pregnancy. The risk factors identified (being mixed race, unemployed, poor self-rated health status, having had TB and partial PTSD) can help in identifying appropriate interventions.
